# Fumarate Hydratase-Deficient Uterine Leiomyomas in Appalachian Women: A Case Series Highlighting Cancer Disparities in Underserved White Populations

**DOI:** 10.7759/cureus.102129

**Published:** 2026-01-23

**Authors:** Smara Sigdel, Srija Pamujula, Waqas Mahmud, Nadim Bou Zgheib, John Diks

**Affiliations:** 1 Pathology, Marshall University Joan C. Edwards School of Medicine, Huntington, USA; 2 Obstetrics and Gynecology, Marshall University, Huntington, USA

**Keywords:** 2sc immunohistochemistry, 2-succino-cysteine, bizarre nuclei, fh-deficient leiomyoma, fumarate hydratase deficiency, hereditary leiomyomatosis and renal cell carcinoma, hlrcc, uterine leiomyoma

## Abstract

Uterine leiomyomas are highly prevalent, yet specific variants, such as fumarate hydratase (FH)-deficient leiomyomas, are rare and clinically significant due to their association with hereditary leiomyomatosis and renal cell carcinoma syndrome (HLRCC). Diagnosis of this subtype relies on identifying unique histopathological features and confirming loss of FH expression or positive S-(2-succino)-cysteine (2SC) staining. This diagnostic challenge is compounded in underserved regions like Appalachia, where health inequities and limited access to specialist care can hinder the management of hereditary conditions. We present a case series of three Appalachian women diagnosed with FH-deficient, 2SC-positive uterine leiomyomas, analyzing their presentation, histopathology, and follow-up within the context of regional healthcare disparities. Histopathological analysis in all cases revealed characteristic features such as focal nuclear atypia, bizarre nuclei, and staghorn vessels. Despite findings warranting genetic evaluation, including significant family histories of cancer in two patients, referral for genetic analysis was inconsistent. These cases illustrate the diagnostic complexity of FH-deficient leiomyomas and underscore the barriers to genetics-based care in Appalachia. Our findings emphasize the critical need for heightened clinical suspicion for atypical leiomyomas, implementation of reflex testing, and improved access to genetic services to optimize outcomes for this underserved population.

## Introduction

Uterine leiomyomas, also known as uterine fibroids, are the most common pelvic tumors in women of reproductive age, affecting more than 70% women globally and serving as the most common indication for hysterectomy [1]. Fumarate hydratase (FH)-deficient uterine leiomyomas represent a rare but clinically significant subset of uterine smooth muscle tumors, as they comprise just 1.6% of fibroids despite being associated with hereditary leiomyomatosis and renal cell carcinoma (HLRCC) syndrome [1,2].

FH is a critical enzyme utilized in the tricarboxylic acid cycle, otherwise known as the Krebs cycle, where it converts fumarate to malate [3]. Sporadic or germline mutations cause fumarate accumulation, which is suspected to cause tumor formation by stabilizing hypoxia-inducible factors (HIF), such as HIF-1⍺ and HIF-2⍺, which promote angiogenesis, proliferation, and survival, via the inhibition of prolyl hydroxylases [3]. Furthermore, a lack of FH causes shunting of cellular metabolism toward glycolysis, initiating the Warburg effect characteristic of cancer cells [3]. Fumarate accumulation further results in histone and DNA demethylase inhibition, promoting tumor progression [3].

HLRCC is a rare autosomal dominant syndrome characterized by a predisposition to smooth muscle tumors and aggressive kidney cancer, driven by germline mutations in the FH gene, consisting of heterozygous germline FH mutations at chromosome 1q42.3-q43 [4]. Clinically, the condition most frequently manifests as multiple cutaneous leiomyomas (75-85% of cases)--small, firm, often painful skin nodules appearing in the second or third decade of life--and early-onset, symptomatic uterine fibroids in nearly 90% of females, often requiring surgical intervention before age 30 [4].

The most critical feature is a 15-20% lifetime risk of developing a distinct, highly aggressive form of renal cell carcinoma (RCC), which can metastasize even when the primary tumor is less than 3 cm. Diagnosis is primarily based on the presence of multiple skin leiomyomas or the identification of a heterozygous FH mutation, with an estimated prevalence of 1 in 200,000 [3,4]. Because the median age for RCC diagnosis is approximately 40 years, early and consistent surveillance via annual abdominal MRI is essential for survival. FH-deficient leiomyomas demonstrate more cellularity, nuclear atypia, and increased mitotic index, making them prone to being misdiagnosed as smooth muscle tumors of uncertain malignant potential and uterine leiomyosarcoma [3]. Key histological features include staghorn vessels, stromal edema creating an appearance similar to that of lung alveoli, large nucleoli with perinucleolar clearing, and hyaline globules in the cytoplasm [2]. 

The most significant risk factor for uterine leiomyomas is race, with black women being disproportionately impacted; others include older age, premenopausal state, nulliparity, obesity, smoking, and alcohol abuse [1]. Further, patients with an annual income <$60,000 may experience more severe symptoms and be less likely to have heard of uterine fibroids earlier [5]. Given the more severe outcomes of leiomyoma in lower-income populations, the prevalence and implications of this diagnosis may be underappreciated in rural, medically underserved populations such as those in Appalachia, in which populations are predominantly white.

Appalachia contains approximately 25 million individuals across 13 states, the vast majority of whom are white (though minority populations continue to increase). Health inequities are prevalent in Appalachia, including those attributed to limited access to care, rurality, lack of quality education, lower income, substance use disorder, and diet [6]. Cancer-related disparities have been observed as well; the incidence and mortality of cancers such as those of the cervix, colon/rectum, and lung/bronchus are higher in Appalachian areas of states compared to non-Appalachian areas, which may be attributed to rurality [7]. Additionally, some research has been conducted into endometrial cancer disparities in Appalachia, with outcomes such as increased incidence of endometrial cancers in Appalachian counties compared to non-Appalachian ones in Kentucky, perhaps attributable to cigarette smoking, obesity, and genetic syndromes [8]. However, until now, no studies have explored the uterine leiomyoma disparities in Appalachia, let alone fumarate hydratase uterine leiomyoma [7].

The infrequency of FH-deficient uterine leiomyomas and unique characteristics, such as a genetic link to HLRCC and hereditary leiomyomatosis, already identify the diagnosis and treatment as a unique challenge. Within the context of socioeconomic disparity in rural Appalachia, these may be compounded by patients’ ability to access sufficient care, seek out genetic testing and family history evaluation, and obtain proper treatment [9]. In this case series, we report upon three white female patients in their forties presenting with uterine leiomyomas. This report highlights previously unidentified patterns in cancer disparity in Appalachia within the context of FH-deficient uterine leiomyomas, paving a path toward more equitable care.

## Case presentation

Case series 

*Case 1* 

**Figure 1:**
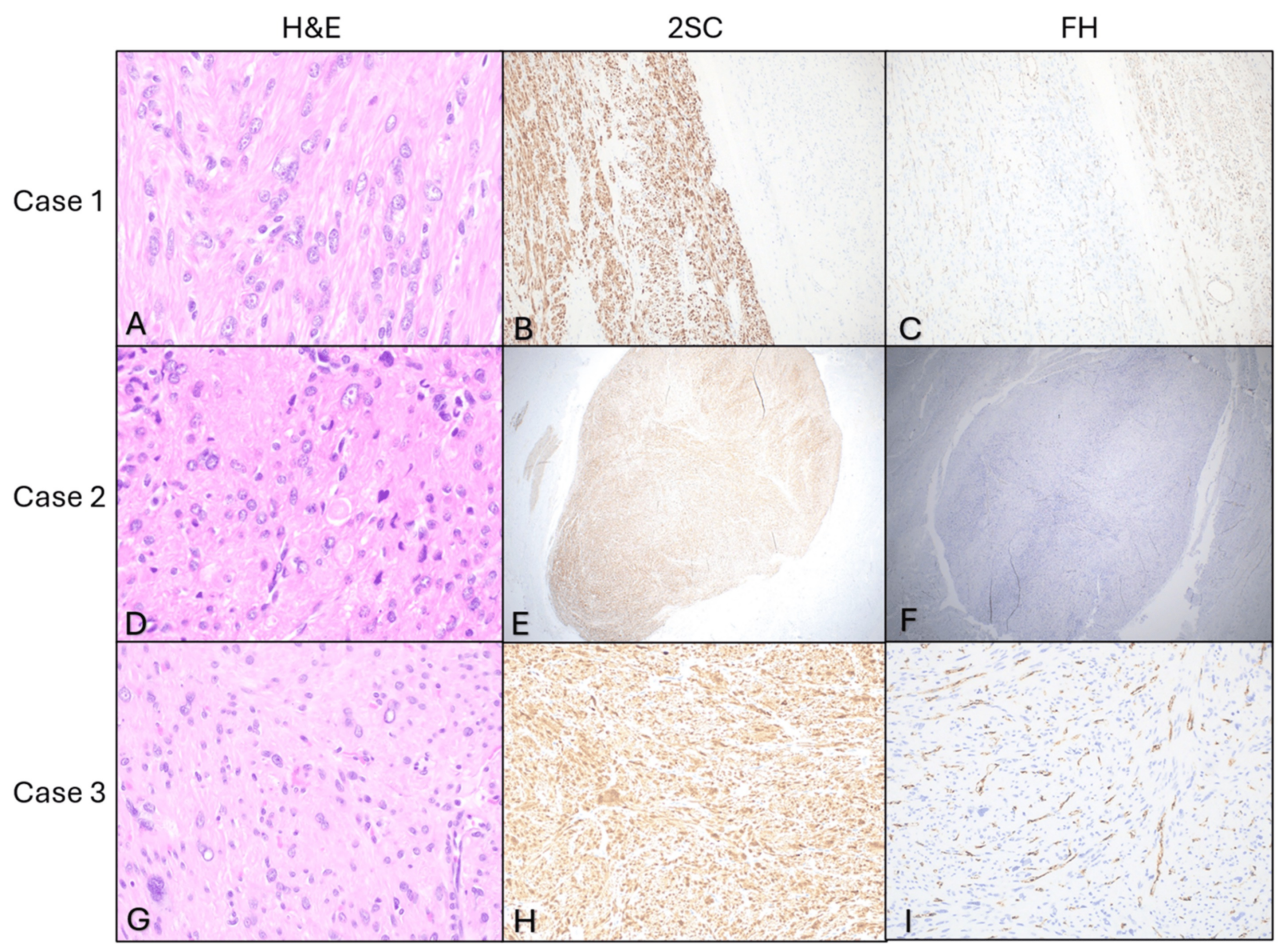
IHC findings in all three cases Case 1: (A) H&E x600, showing eosinophilic nucleoli with peri-nucleolar halos. (B) S-(2-succino) cysteine (2SC) IHC study x100, showing strong and diffuse staining (block-like) in cytoplasm and nucleus in tumor cells. (C) FH IHC x100, showing complete absence of immunoreactivity in tumor cells. Case 2: (D) H&E x600 showing ovoid nuclei and prominent eosinophilic nucleoli surrounded by peri-nucleolar halos and intracytoplasmic rhabdoid inclusions. (E) 2SC IHC x20, showing strong and diffuse staining (block-like) in cytoplasm and nucleus in tumor cells. (F) FH IHC x20, showing complete absence of immunoreactivity in tumor cells. Case 3: (G) H&E x400, showing intranuclear pseudo-inclusions and multinucleation. (H) 2SC IHC x200, showing strong and diffuse staining (block-like) in cytoplasm and nucleus in tumor cells. (I) FH IHC x200, showing complete absence of immunoreactivity in tumor cells. H&E: hematoxylin and eosin; IHC: immunohistochemical; FH: fumarate hydratase

A 45-year-old white female residing in Appalachia with a family history of brain cancer presented with an abdominal mass. The patient had a history of past narcotic use, was a former smoker, and currently utilized smokeless tobacco. Relevant current medications include atorvastatin, lisinopril, metoprolol tartrate, Ozempic, and suboxone (Table 1). After undergoing total abdominal hysterectomy and bilateral salpingo-oophorectomy (TAHBSO), pathology revealed a large, 16 cm intramural leiomyoma and two subserosal leiomyomas with atypia, characterized by nuclear atypia but no increased mitotic activity or necrosis (Table 2). Immunohistochemical staining demonstrated loss of FH expression and S-(2-succino) cysteine (2SC) overexpression, confirming FH-deficient leiomyoma (Figure 1). The patient was referred to genetics for further evaluation. 

**Table 1:** Clinical and gross pathological findings in patients with FH-deficient leiomyomas FH: fumarate hydratase

Case	Age (years)	Family History	Substance Use History	Surgery	Gross Pathological Findings
Case 1	45	Brain cancer (father)	Past narcotics use	Total abdominal hysterectomy and bilateral salpingo-oophorectomy	Enlarged uterus 17-18 cm
	-	-	Former smoker	-	3 low-density defects in the left kidney, too small to characterize but likely cysts
	-	-	Smokeless tobacco	-	19 x 14 x 19 cm mass arising from the pelvis (suspected to originate from the right ovary)
	-	-	-	-	Urinary bladder
Case 2	45	Breast (sister), cervical (mother), non-Hodgkin lymphoma (brother)	Past narcotics use	Robotic-assisted total laparoscopic hysterectomy with bilateral salpingo-oophorectomy and IUD removal	Leiomyomata and adenomyosis
	-	-	1/2 pack of cigarettes or more/day in the last 30 days	-	Adhesions involving the anterior surface of the uterus and bladder
	-	-	-	-	Exophytic fibroids
	-	-	-	-	Enhancing confluent
Case 3	41	Unknown	Unknown	Hysterectomy	7 mm hypodensity lateral midpole right kidney, too small to characterize, but likely a cyst
	-	-	-	-	2.3 cm mildly irregular, peripherally enhancing hypodense structure within the right adnexal region with a small amount of adjacent free fluid, likely a ruptured physiologic cyst
	-	-	-	-	Contracted appearance of the gallbladder
	-	-	-	-	Numerous heterogeneous enhancing confluent masses in the pelvis

**Table 2:** Morphological and IHC findings in patients suspected of having FH-deficient leiomyomas IHCL immunohistochemistry; FH: fumarate hydratase; 2SC: S-(2-succino) cysteine

Cases	Leiomyomas Detected	Morphology	FH	2SC
Case 1	Intramural leiomyoma (x1) 16 cm in size; Subserosal leiomyoma (x2) 0.5 - 2 cm in size	Atypia in subserosal leiomyoma without an increase in mitotic activity or necrosis, eosinophilic nucleoli with peri-nucleolar halos	Neg	Strong diffuse staining
Case 2	Multiple uterine fibroids; the largest was 15 - 16 cm in size	Focal nuclear atypia, bizarre nuclei, ovoid nuclei, prominent eosinophilic nucleoli, peri-nucleolar halos, and intracytoplasmic rhabdoid inclusions, alveolar edema, staghorn vessels without necrosis, 0 mitoses/10 HPF (high-power fields)	Neg	Strong diffuse staining
Case 3	Nodular uterine leiomyomas (x3); the largest was 1.6 cm	Intra-nuclear pseudo-inclusions and multinucleation	Neg	Strong diffuse staining

*Case 2* 

A 45-year-old white female from Appalachia presented with a pelvic mass and underwent robotic-assisted total laparoscopic hysterectomy and bilateral salpingo-oophorectomy (BSO) with IUD removal. Relevant medications included aspirin and carvedilol. Past substance use included narcotics, and ½ pack/day smoking tobacco use in the last 30 days (Table 1). Pathology revealed multiple fibroids, with one showing focal nuclear atypia, bizarre nuclei, and staghorn vessels (Table 2). Immunohistochemical staining confirmed FH-deficient leiomyoma (Figure 1). Family history was notable for breast, cervical, and non-Hodgkin lymphoma cancers. This patient’s presentation with rapidly enlarging symptomatic fibroids is consistent with the more aggressive phenotype described in the literature for FH-deficient cases.

*Case 3* 

A 41-year-old white female presented with abnormal uterine bleeding and pelvic pain (Table 1). Hysterectomy specimen contained three nodular uterine leiomyomas (largest 1.6 cm) (Table 2). Immunohistochemistry confirmed loss of FH and 2SC positivity, indicating FH deficiency (Figure 1). Family history and social determinants of health were less documented.

The laboratory profiles for Case 1 and Case 2 are largely unremarkable, with hematological and metabolic markers, such as WBC, calcium, and albumin, falling within standard reference ranges. However, Case 2 shows a borderline low hemoglobin level (11.8 g/dL). In contrast, Case 3 presents with more significant deviations, including mild anemia (hemoglobin 10.5 g/dL) and a slightly elevated creatinine level (1.4 mg/dL), which could indicate a mild reduction in renal function. Platelet counts and glucose levels remained stable across all three cases (Table 3).

**Table 3:** Clinical laboratory tests for the three cases

Laboratory Test	Case 1	Case 2	Case 3	Reference Range
WBC (x10^3^)	8.8	5.2	4.8	4.5–11.0
Hemoglobin (g/dL)	13.2	11.8	10.5	12.0–16.0
Platelets (x10^3^)	245	198	165	150–450
Creatinine (mg/dL)	0.9	1.1	1.4	0.6–1.2
Glucose (mg/dL)	98	105	112	70–99
Calcium (mg/dL)	9.4	9.1	8.9	8.5–10.5
Albumin (g/dL)	4.2	3.8	3.5	3.5–5.0

## Discussion

**Figure 2:**
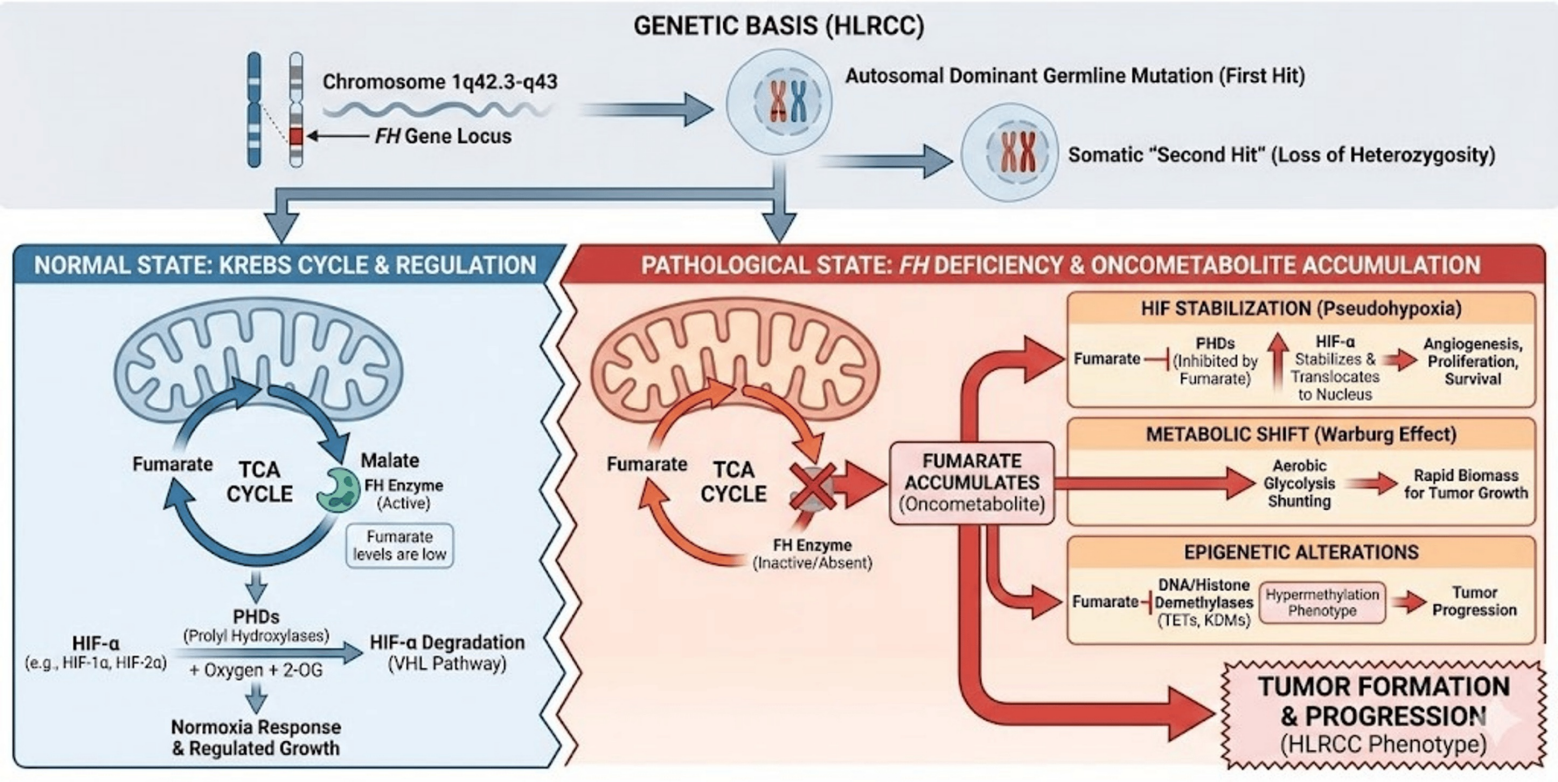
Biochemical and genetic pathogenesis of HLRCC-associated FH deficiency Schematic representation of the molecular consequences of fumarate hydratase (FH) deficiency (Top) Hereditary leiomyomatosis and renal cell carcinoma (HLRCC) follows an autosomal dominant inheritance pattern with a germline mutation in the fumarate hydratase FH gene (1q42.3-q43), followed by a somatic "second hit" (loss of heterozygosity) that inactivates the remaining wild-type allele. (Bottom) Loss of FH enzymatic activity disrupts the tricarboxylic acid (TCA) cycle, preventing the conversion of fumarate to malate. The resulting intracellular accumulation of fumarate acts as an oncometabolite that: (1) competitively inhibits prolyl hydroxylases (PHDs), stabilizing hypoxia-inducible factors (HIF-⍺) to induce a pseudohypoxic state driving angiogenesis and cell survival; (2) forces a metabolic shift toward aerobic glycolysis (Warburg effect); and (3) inhibits DNA and histone demethylases (TETs, KDMs), leading to a hypermethylator phenotype that promotes tumor progression. The infographic was generated using Google Gemini (Advanced), which utilizes the Imagen 3 model for image generation. The figure was created based on a specific prompt and conceptual design provided by the authors to visually synthesize the biochemical pathways described in the text.

HLRCC is an autosomal dominant syndrome driven by germline mutations in the FH gene (1q42.3-q43), which encodes the tricarboxylic acid (TCA) cycle enzyme FH [3]. Tumorigenesis typically follows Knudson’s two-hit hypothesis, where a somatic "second hit" silences the wild-type allele, causing a loss of enzymatic activity and the subsequent accumulation of intracellular fumarate. Acting as a potent oncometabolite, excess fumarate competitively inhibits ⍺-ketoglutarate-dependent dioxygenases, most notably prolyl hydroxylases (PHDs). This inhibition prevents the degradation of hypoxia-inducible factors (HIF-1⍺ and HIF-2⍺), stabilizing them to induce a "pseudohypoxic" state that drives angiogenesis and cell survival [3]. Concurrently, mitochondrial dysfunction necessitates a metabolic shift toward aerobic glycolysis (the Warburg effect), while the inhibition of histone and DNA demethylases induces a hypermethylator phenotype, collectively fostering a pro-oncogenic environment (Figure 2) [3].

FH-deficient uterine leiomyomas are a crucial diagnostic consideration in relatively young women presenting with multiple or atypical leiomyomas, enlarged uteri, or a family history suggestive of HLRCC. Distinguishing these from leiomyosarcoma or other atypical variants is vital for management, genetic counseling, and renal cancer surveillance [2]. FH-deficient uterine leiomyomas commonly may be present singularly or in multiples, and removal through hysterectomy is largely effective. Gross pathology may include homogenous, white, soft, amorphous tissue lacking nodules or whirls seen in other uterine leiomyomas. Histologically, features may include increased cellularity compared to other uterine leiomyomas, chain-like tumor cell arrangement in collagenous matrix, eosinophilic nucleoli and/or cytoplasmic inclusions, and staghorn vessels. Nuclear atypia may or may not be present, including multinucleation, pseudo-inclusions, and mitotic figures [10].

Female patients with HLRCC tend to have large uterine fibroids that appear earlier than with typical uterine leiomyoma. Uterine leiomyomas may present as the initial early diagnosis of HLRCC, and patients and family members may be encouraged to pursue additional testing, screening, and surveillance for renal and skin tumors [10]. Detection of uterine leiomyomas, even without any cutaneous or kidney abnormality, but especially with a family history of uterine fibroids, may be key to diagnosing HLRCC early [11]. As such, genetic counseling may be advisable given the association with HLRCC and hereditary leiomyomatosis. In a case series by Alkhrait and colleagues, out of five patients with FH-deficient uterine leiomyoma, one developed renal malignancy a year following hysterectomy (Table 4) [4]. It has been highlighted that, granted that somatic mutations may also result in FH-deficient uterine leiomyomas, explaining why FH-deficient leiomyoma prevalence is about 1.6% while the frequency of HLRCC is about 1 out of every 2668 individuals [3]. S-(2-succino)-cysteine (2SC), which concurrently accumulates with fumarate in FH deficiency, is sensitive for HLRCC-associated renal cell carcinoma, especially if concurrent with a negative stain for FH, but is not commercially available nor routinely used. It is thus suggested that 2SC positivity may allow for more sensitive detection of uterine leiomyomas as well [2,12]. Management of FH-deficient leiomyomas, therefore, requires typical surgical removal and/or hormonal therapy in addition to surveillance for genetic counseling and surveillance for renal cell carcinoma, inclusive of annual abdominal imaging (Table 4) [3]. 

**Table 4:** Brief literature review of FH-deficient leiomyomas FH: fumarate hydratase

Year	Author	Number of patients	Tumor type	Important findings
2016	Harrison WJ et al. [12]	5	FH-deficient uterine leiomyoma	Case report detailing the differences between sporadic and syndromic settings of FH deficient uterine leiomyoma
2016	Miettinen M et al. [10]	86	Fumarase deficient uterine leiomyoma	Highlighted molecular features and IHC markers in fumarase deficient uterine leiomyomas
2023	Alkhrait S et al. [4]	5	FH deficient uterine fibroids	Case report describing diagnosis, clinical presentation, and early management
2023	Shero N et al. [11]	1 (5 case reports from literature review)	HLRCC	Case report stressing the importance of genetic testing for FH deficiency after hysterectomy in patients with suspected HLRCC
2024	Kamboj M et al. [2]	5	FH deficient uterine leiomyoma	Case report detailing the differences between FH deficient uterine leiomyoma, leiomyosarcoma, and other malignant spindle cell tumors
2025	D’Indinosante M et al. [3]	NA – literature review	FH deficient tumorigenesis	Review of basic features of FH deficient leiomyoma, including management and suggestions on future treatment development
2025	Khan A et al. [9]	1	FH deficient uterine leiomyoma	Diagnostic challenges between FH deficient uterine leiomyoma and malignant conditions

However, as shown in this series, women in Appalachia may face delays at multiple steps: presentation, diagnosis, immunohistochemistry, and access to genetics services [7]. Even access to gynecologists proves to be a challenge; in West Virginia, new gynecologic oncology patients travel more than one hour to access West Virginia University’s Mary Babb Randolph Cancer Center, which is located at the largest tertiary cancer center in the state [13]. Perhaps as a result, West Virginia’s incidence of and mortality from gynecologic cancers is higher than that of the US as a whole [13]. Regarding FH-deficient uterine leiomyoma, where genetic analysis and counseling should be pursued, yet another obstacle arises. Cohen and colleagues write that genetics providers primarily work in large academic medical centers, causing limited access to genetic testing, prompted by the specialist [14]. This places the burden upon primary care providers, who have already demonstrated a concern regarding their knowledge of genetic disorders, causing them to refer the patients to a specialist [14]. As a result, genetic services become further overwhelmed and delayed while also requiring families to accommodate long waits and laborious travel, all the while being unsure as to whether insurance will cover the assessment [14]. In totality, limited access to initial care and later genetic services may prove to be deleterious to the outcome of the patient.

Furthermore, the history of smoking tobacco and narcotic use and use of medications with indications for diagnoses like metabolic diseases and hypertension in both case 1 and case 2 underscores regional comorbidities that may contribute to leiomyoma prevalence and outcome. Twenty-one percent (21%) of West Virginian adults smoke cigarettes, and in 2017, West Virginia had the highest per capita overdose fatality rate [15,16]. It is particularly well-known that smoking tobacco increases the risk for numerous cancers, with 37.8% of cancer deaths in West Virginia associated with smoking [15]. Appalachian patients may therefore benefit from public health education about substance use and tobacco smoke risk and abuse [17]. In the same breath, further education regarding cancer heritability may encourage earlier information seeking and thus result in earlier diagnosis of cancer, despite challenges regarding genetic testing [18]. In essence, improved health literacy about heritable uterine cancers and preventable risk factors may prove to be beneficial in disadvantaged areas of Appalachia (Table 5).

**Table 5:** Brief literature review of health conditions in Appalachia

Year	Author	Number of patients	Tumor type	Important findings
2011	Paskett ED et al. [7]	NA	NA	Cancer-related disparities in underserved white populations in Appalachia
2020	Johnson MS et al. [8]	Population-based cohort	Uterine Corpus Malignancies	Uterine corpus malignancy-related disparities in Appalachian populations
2023	Driscoll DL et al. [6]	NA – literature review	NA	Review of health outcomes for prevalent diseases in Appalachia
2024	Cohen ASA et al. [14]	1083	NA – rare diseases	A study that correlated access to genetics-based counseling, wait times, and health outcomes
2025	Whitfield K et al. [13]	Population-based cohort (1097)	NA	A study focused on relating geographical locations and health outcomes for patients with gynecologic oncology needs in West Virginia

The cases presented in this report highlight the diagnostic complexity of FH-deficient uterine leiomyomas and the health disparities and potential deficiency of complete care in Appalachia. In all three cases, histopathological findings pointing to FH-deficient uterine leiomyoma indicated the pursuit of an immunohistochemical stain. However, while all three cases also demonstrated 2SC overexpression, which should indicate genetic screening, a mere referral to genetics was only noted in one case. As 2SC positivity is sensitive for HLRCC, it is imperative that these patients receive prompt genetic screening. These cases, therefore, demonstrate the challenges of genetic risk assessment, significant because coordinated genetic care and follow-up is limited in the underserved region, as none of the three patients had a genetically confirmed diagnosis of HLRCC due to the absence of genetic testing results (Table 5) [7]. 

Appalachia’s population is predominantly white, rural, and economically challenged, with a higher burden of cancer and poorer health care infrastructure compared to the U.S. at large. This environment exacerbates challenges in recognizing and managing rare syndromic lesions that require sophisticated diagnostic and follow-up resources. Our case series underscores the need for regionally targeted educational and diagnostic strategies, including the adoption of reflex FH testing for unusual leiomyomas and improved genetics referral pathways (Table 5) [7].

## Conclusions

This series demonstrates the consistent under-recognition of FH-deficient uterine leiomyomas in Appalachian women. The cases highlight the need for increased clinical suspicion, better laboratory support, and improved access to cancer genetics in underserved white populations to reduce disparities. Regional initiatives, through partnership, education, and infrastructure investment, are necessary to close the gap in cancer outcomes for Appalachian women at risk of hereditary cancer syndromes.
